# Book Reviews

**Published:** 1815-11

**Authors:** 


### CRITICAL ANALYSIS
### OF RECENT PUBLICATIONS
IN THE
### DIFFERENT BRANCHES OF PHYSIC, SURGERY, AND MEDICAL PHILOSOPHY.

---

*Minutes of Cases of Cancer and Cancerous Tendency successfully treated;* by Mr. SAMUEL YOUNG, Surgeon, Author of a Treatise on Cancer, and other Surgical Works; with a Prefatory Letter addressed to the Governors of the Middlesex Hospital, by SAMUEL WHITBREAD, Esq. M. P. London. Cox and Co. 8vo. pp. 150; 1815.

IT may not be known to some of our readers, that, about thirty years ago, Dr. Denman remarked, how desirable it would be if a charity should be formed entirely for the relief or cure of cancer. Soon after this, the governors of the Middlesex Hospital received, from an unknown source, £2000, and it was not discovered till after the death of Mr. Whitbread, father of the late Samuel Whitbread, Esq. whence so generous a benefaction was derived. Though the cancer ward of the Middlesex Hospital has not furnished any considerable improvement in the treatment or information concerning the nature of the disease, yet this must be imputed to the difficulty of the subject, and not to any inattention in the medical officers of that house.

Mr. Young, as we have remarked in pages 384–5 of this number, has several years ago given his opinion on cancer. Since that time he seems to have directed his whole attention to the subject, and now offers the result of his labours under the powerful recommendation, we might say under the sanction, for the truth of all he states, of no less a name than the late much-lamented character, whose industry and accuracy evinced itself in whatever he undertook.

" On the 15th of September last, (says Mr. Whitbread, in a Letter addressed to the Governors of the Middlesex Hospital) I met Mr. Young, at the house of Dr. Penrose, at Hatfield; and, having received from the latter the most positive assurance, that the plan could not produce any mischief, and afforded a prospect of relief, the experiment was decided upon; and on the next day it commenced, at the residence of the patient, within a short distance of the town of Bedford, in the presence of Dr. Penrose and myself. On the same day, Anne Wildman, whose case is amongst the most remarkable of those detailed by Mr. Young, was brought from the Bedford House of Industry, and after minute examination by Dr. Penrose, subjected to the same process.

" From

"From that period, Mr. Young has resided in Bedford and its neighbourhood; and several patients afflicted with cancer, or diseases of that tendency, have been placed under his care.

"The minutes of the cases are drawn up by Mr. Young; and the attestations and opinions of the medical men, and others who had known the origin, or seen the progress of any particular case, or had been called in to decide upon the effect of the treatment, are given in their own words.

"The very remarkable case wherein the practice was so promptly adopted by Mr. Macgrath, of Biggleswade, from having seen its application under the hands of Mr. Young, is described by Mr. Macgrath: the value of whose testimony is known to an extensive circle.

"I thought it might be of some service to the cause of humanity, to see with my own eyes some of the cases; and I have been more than repaid by witnessing the progressive amendment of the patients, and by hearing from time to time their joyful declarations, that they were greatly relieved from the torment of their previous pain.

"Where Mr. Young has introduced my name, he has faithfully described my impressions."

Mr. Young commences with a general account of his plan of operations.

"Pursuing (says he,) the same train of facts, an account of which was published in 1805, under the title of 'An Inquiry into the Nature and Action of Cancer, in order to establish a Principle of Cure by Natural Separation,' where it was proved, that the disease can only exist under a previously altered and morbid accumulation of structure; but, considering the necessarily confined application of such a principle of cure, in a disease so complicated, so variously acting, so indefinite in extent, and so often involving parts necessary to life itself; the idea of a more extended effort for the removal of the disease by *mechanical pressure* suggested itself; and so far back as the year 1809, in two cases, it was successfully employed.

"The principle of this practice was found in the obvious facts, which nature is constantly presenting in the operations of the animal œconomy, viz. the removal of parts by absorption, and particularly under the excitement of pressure. It is true, such facts were presented through the medium of disease, but the principle was the same, and only required another direction to obtain beneficial and healthy results.

"If by diseased tumours the brain can be absorbed, the bones of the skull removed, or the testis in hernia congenita obliterated, to reverse the powers of absorption seemed feasible. If, by diseased pressure, healthy structure could thus be removed, why not imitate such pressure, and take advantage of such powers, by directing them to beneficial purposes, and destroy in turn morbidly formed parts or tumours, by the very means with which Nature has furnished them, for the destruction of natural structure?

"By such morbid pressure arterial action also is suppressed. Why then should a tumour of the neck, for example, be suffered to proceed in all the licentiousness of disease, destroying on the one hand by its pressure the natural circulation of parts, and on the other exciting the absorption of natural by unnatural structure; when the same pressure, employed on its own surface, would obstruct its growth, and cause its own absorption? Under this impression, and on the principle that mechanical pressure resists vascular action, and excites the absorbents, it was adopted as a mode of cure for the removal of cancer, and other diseases dependent on previously altered and morbidly accumulating structure. One might enter largely on the more minute operations of this process, in the prevention and restoration of diseased structure; but the present object is simply to give a brief statement of leading facts. There are, however, many curious and important truths, connected with and illustrative of the nature and history of diseases of this class, the detail of which shall be given at some future opportunity.

"The means generally employed to effect the pressure as stated in the following cases, have been plaster-straps, sheet lead forming shields of various thicknesses, tin plates, linen compresses, and the use of appropriate rollers.

"The strength of the application of the pressure has been progressive, commencing in most cases with the use of the straps only; in some by single, and in other cases by double layers. The force of their application controlled in each instance by the existing circumstances, and the sensations of the patient.

The plaster should be uniformly smooth; and in the application of the straps it is of the first importance that all wrinkles should be avoided; that an equal surface of resistance should be given. In the direction of specific pressure on a diseased part, all sort of partial stricture must be avoided, according to the common principles of surgery, which may be illustrated by the now common treatment of an ulcerated leg, after the admirable plan of Baynton. Here the ulcer of the leg is specifically compressed, although general pressure is also given to the limb, by the use of the roller. So a scirrhus of the breast may be specifically compressed, by the use of the pressure plates, and the adjustment of the linen compress, including at the same time a general pressure of the whole."

The rest of the work consists of cases minutely detailed and amply authenticated. As we cannot be expected to offer any opinion on a subject so new, all that will be required of us in the present state of the inquiry is to furnish sufficient evidence, that Mr. Young is entitled to a candid hearing. We have selected two cases, not as more pointed than the rest, but because the disease was seated in differently constructed parts and in different sexes, and because the interest of both much encreased by the relation of the patients themselves.

"*The Case of the Lip of Mr. Lea, from Flamstead,* [*written by himself.*]

*himself.*]—'First came a small sore, and called a wart. Tried means to kill it: but all to no purpose. It has been coming to what it now is for this four years. The symptoms are, continual pain, otherwise shooting. Grew to a scurf, and then came off; and when off, underneath was a seedy root like to a cauliflower, and then grew again in a little time.'

"This statement, though not explicitly entering into detail, sufficiently shews the active nature of the disease, and the progressive morbid change, that has been going on in the structure of the lip. For the increase of the tumour, it is to be noted, has been from the enlargement of its base, and consequent involving the natural structure of the lip.

"Upon questioning the patient what he means by growing to a scurf, and then coming off, it appears that the whole projecting surface of this cancerous mass, or wart, or excrescence, arising from an actively diseased and morbidly changed mass leading to cancer, has been in a state of ulceration for a length of time. That the different surfaces so exposed have shortly died, turning to a black, smooth, and rather polished substance. That these partial disengagements have given a temporary strength of growth to the part, which has always ended in a succession of superficial slough. The time between the operations of nature being from four to five weeks.

"The incrustations, when thrown off, have been about two lines in thickness, retaining the form of the excrescence they covered; and the surfaces then exposed have had a hard sort of structure, like white cauliflower. These in turn, after a short growth, became of a dark colour, till they were changed to the uniform black incrustations described. So that, while the base of this tumour has been progressively increasing in intimate morbid connection with the structure of the lip, sloughing and separation, with partial growth of its surface, have been going on in constant succession; strongly exemplifying the nature of such diseases, the strength they gain by mere partial destruction, and the consequent mischiefs of all partial removals.

"During the four years progress of the disease, the patient consulted five professional gentlemen; but no remedy was found to check its advance. Caustics and leeches were applied to the lip, under the care of one surgeon, without avail; and Lea's health at length became so much affected from continual pain, as to require the advice of an eminent physician: fresh applications were ordered to the lip, as well as general remedies; but still the local and constitutional symptoms continued in an aggravated state of advancement.

"For the last six months, and till within a week or two of his present application, the patient has been exposed to a severe caustic treatment, under the direction of some unprofessional person. This only produced insufferable torture, and an aggravated state of the disease in all its circumstances, both as to the degree, duration, and extension of pain, as well as the rapid change of of the lip towards disease,—the whole of which is now little short of a complete scirrhus.

"From the margin of the black film, foul ulcers run inward into its substance, and a putrid effluvia is emitted, which is offensive at some distance. This last symptom was particularly brought to my notice, by the man who accompanied the patient to Bedford.

"The patient complains of a constant dry burning heat of the skin, particularly affecting the right arm and hand, and more especially at his accustomed hour of rising, and about four o'clock in the afternoon.

"The following was ordered—

℞. Calomel. ppt. gr. iv.
Pulv. fol. Cicutæ—Ant. a. a. gr. ij.
Opii pulv.—fol. Digitalis a. a. gr. j.
Conf. Opiat. q. s. ft. Pil. N. ij. h. s. s.

℞. Pulv. Jalapii ʒſs.

Vini Ant. gtt. xv.

Sp. Ammon. c. ʒſs.—Lavand. c. ʒi.

Aquæ Menthæ sat. ℥iſs. M. ft. haustus cras mane sumendus."

Other remedies were used, particularly the arsenical preparation of Dr. de Valangin. We do not mention this to lessen the value of Mr. Young's method by pressure, because it is well known that they have been frequently and ineffectually tried in cancer.

"March 14.—The lip quite healed, and restored to its natural state. A very active plan of pressure has been continued up to yesterday. The patient returns home, with directions of great caution to avoid the severe winds now prevalent, all excess in living, and to pursue the alterative and mineral solution plan, under the usual restrictions, for a week or two longer.

"March 19.—Received a letter from the patient, wherein he mentions, that his lip is as smooth and level as ever it was in his life; that some little itching on the nose and lip remain; but that he supposes the medicine will stop it.

"March 30.—Another letter has been received from the patient, wherein he states, that he continues comfortable; only a little itching and tingling remain.

"May 26.—This day brought the satisfaction of another letter from the patient. He expresses himself in strong terms, and adds, 'My lip continues comfortable.' No mention is now made either of itching or tingling; and thus this case has ended in the restoration of perfect health.

"*First Letter to Mr. Whitbread.*

"Hon. Sir, *March* 19, 1815.

"I received your's this day respecting my lip, which, I am happy to inform you, is, I hope, cured; at which I am perfectly happy, and return my friends thanks, next to the Lord.

"Sir, you had seen it at Bedford, in a bad state. Now my lip is level and smooth as before it came on it. Sir John Sebright has seen, and he was surprised. I wished to have shewn it to you, only you had left Bedford, and I in a weakly state, otherwise I should have took the journey to London; and, if you wish to see me, will now. Your obedient humble servant,

*Flamstead.* Wm. Lea."

"*Second Letter to Mr. Whitbread, in answer to a particular inquiry respecting the commencement of the disease, and the treatment of it previous to Mr. Young's seeing it.*

"Hon. Sir,

"I received your kind letter, concerning my lip. Thank God, it is much the same as when I left Bedford, five weeks ago. Thank you for your goodness of recommending me to Mr. Young; and, please God, I hope it will remain well. The first of its coming, five years ago; it came a small scurf on my lip, and it was called a wart. I applied means for a wart, but to no purpose, for a year or two. Then Dr. Chase, of Luton, took it in hand, and applied caustics and leeches to the lip for some time, to no purpose. He did not persevere in it now, as it was called a wart. He did not say what it was. Then we stopt. Then Mr. Somers had it in hand a considerable time, and no better. Then he had Doctor Penrose to see it. He give Dr. Somers advice how to treat with it, and give me medicines and applied something to the lip, and got no better. Then wish me to have Ashley Cooper's advice; but I did not go. Doctor *Winkfield* wished me to have it cut out; but I did not agree to it. Dr. Warren, Surgeon now to the Duke of York's Asylum, Chelsea, saw it two or three times. He wished me to have the best advice. He said it was a cancerous substance. After all this, we heard of a woman at Westoning, Bedfordshire, that cured cancers. I applied to her, as it grew so painful and bigger, and she applied a burning caustic, which caused me a deal of pain, and got no better. She called it a dry cancer.

"Then Mr. Whitbread and Sir John Sebright recommended me to Mr. Young, and he gave me a diligent attendance, and now, please God, it remains comfortable, and I hope will continue so. Mr. Young deserves merits and thanks; likewise you, gentlemen.

"This is the only information I can give, Sir. I wish you to see the lip, as you saw it in bad state. I expect to be in London in a short time; then I shall take the liberty to call on Mr. Whitbread.

"Excuse my blunt way of explaining myself.

I remain,

Your obedient humble servant,

*April* 20, 1815. Wm. Lea."

"N.B.—Mr. Lea afterwards came to London, and called on Mr. Whitbread. His lip appeared in a sound state."

The next case we shall notice is the last in the book, and if the cure is not complete, enough has been done, we conceive, ceive, to make it the duty of every surgeon to attempt the same in these deplorable cases.

"*Case of Mrs. Jennings, of Harlington, in Bedfordshire*, aged 56; visited at Harlington, on Tuesday, Dec. 6th; first medical attendance at Bedford, Dec. 16, 1814.

"Whether considered by the symptoms that attend it, the various parts affected, the extension of those parts, or the advanced and different stages of progress of the disease, this case presents one of the most extraordinary instances of complication that ever existed, or can well be imagined. All the circumstances that have ever been enumerated as attendant on cancer, may here be seen at one view; and are consolidated in this one individual case.

"The remains of the left breast, which was the first affected, is so disfigured, and lost by frightful puckerings, as scarcely to leave a trace of its original form. A portion of the upper part only is left a partially soft and insulated mass. Two thirds of this, however, is scirrhous, and deeply drawn into fixed and immovable folds. The nipple is lost in a deep chasm of stony ulceration, which runs to the ends of the scirrhous folds under the arm. From this the disease spreads widely on the chest, in little holes, or scirrhous ulcerated knobs, running one into another, and passing by a chain of deeply diseased and ulcerated structure, to the remains of the other breast, involving the whole in one continued cancerous sore.

"The right breast presents morbid change exactly similar to the left, only that the decay or ulceration at the centre has not proceeded to such a length; and the retracted mouldered remains of what might formerly have served as the base of the nipple, may still be seen in the deep fissure formed between the puckered scirrhus and ulcerated masses.

"Except a small cushion-like protuberance, (the only thing left of natural structure,) the whole of what remains of this breast is also firmly drawn in, and fixed to the side.

"The integument of the chest is one immovable plate of disease, thickly studded by hard glandular knobs. On either side of the throat a diseased mass of absorbent glands exists; but on the right side they are considerably the largest.

"Both arms have been long and deeply affected; but the glands of the left axilla are those that exhibit longer-established morbid alteration. The right arm and breast, notwithstanding, have always been the most painful. The right arm in particular, at this time, is greatly enlarged, and from the elbow upwards is hard and tense. The left arm also is considerably increased beyond its natural size.

"Extending down each side, from the sores of the breast, the integument is much diseased, full of knots, drawn up in folds, or quite changed, and lost in scirrhous masses. But the morbid alteration of the left side is the more strongly marked. Here the diseased integument is drawn and puckered in a most extraordinary manner, manner, affording the appearance of a claw firmly grasping the side.

"On each side, from the breast to the axilla, the whole space is occupied by hard knobs, deep scirrhous puckerings, and folds in a most curiously confused state. On the left side it is inveterately marked. One fissure or fold is at least an inch in depth, and four or five inches in length, at the bottom of which a deeply imbedded scirrhous ridge of glands may be felt, leading to the back of the shoulder, where also there is a large mass of diseased glands. This fold is in a state of partial ulceration.

"On the inner side, within two inches of the right fissure of the breast, there is a remarkable deep pea-like sore, which forms the centre of a scirrhous circle, about an inch in diameter, which is remarkably diseased, and of impenetrable hardness. This sore, it is supposed, was occasioned by the application of a leech. All the ulcerated and contiguous parts are discoloured; mostly of a livid purple hue, and of stony hardness.

"The sores have been dressed with some simple ointment on linen, twice a-day; and large bleedings have frequently, indeed commonly, attended their removal. On the day I attended at Harlington, upon the removal of a small part of the dressing, a sudden gush of blood followed; which, Mrs. Jennings observed, was trifling, when compared with what often took place. The character of the discharge is that of a thin fetid ichor.

"In recapitulating this case, as it now presents itself, it may be observed, that the whole of the chest is cancerously diseased, including both breasts, extending down the integuments, almost to the abdomen, and comprehending on either side a large mass of surface, reaching from the breast to the axilla.

"In the axillæ, the glands are so deeply diseased, that their morbid extent is beyond the power of any examination to define; and so complicated, as to defy every attempt at description.

"The left breast is almost obliterated, and the nipple and surrounding parts degenerated into a deep destructive sore; from this, reaching over the sternum, and including the other breast, is one sore and cancerous mass. Both arms are swelled, hard, and painful. The neck almost fixed by diseased glands. The circumstance even of the bleeding cancer is not wanting to fill up the catalogue of those evils which the experience of centuries has selected as attendant symptoms of the disease; selected, however, from a great variety of cases, and, fortunately for suffering humanity, but rarely, if ever, before found centred, at the same time, in one individual. But it is a great consolation to add, under the pressure of such a mass of foreboding evils, that extreme or even violent pain has been hitherto unknown. The state of the arms has occasioned the greatest uneasiness. At this time, Dec. 16th, considerable fever and general irritation exist; and, as this state was still more aggravated a few days back, it is not thought advisable at present to commence the plan of pressure. The following alterative has been ordered:

 ℞. Calomel.

℞. Calomel. ppt. gr. vj.
Pulv. Ant. gr. x.—fol. Digitalis gr. viij.
Ex Sarsæ ʒ ſs. M. ft. Pil. N. 8.—Capt. j. h. s.

"The saline draughts to be taken occasionally through the day, and the foot warm-bath at night, and other ordinary remedies employed. It may be proper to remark, that, when I made my first visit to Harlington, Mr. Green, of Woburn, the medical gentleman attendant in the family, was present, who declared the case very much worse, and the sore greatly extended, since he had last seen it. He also represented to me that no regular attendance had been given, as the case had been considered quite out of the reach of relief. That for some time steel had been exhibited under the direction of Mr. Cooper, and continued till the fever it induced obliged its suspension.

"In the hopeless state of the case I quite agreed; but strongly urged the adoption of a plan of pressure upon two grounds: first, for the suppression of the hæmorrhage; and secondly, to suspend for a while, if not permanently to check, the rapid progress of the disease, which was fast advancing to involve the whole surface of the chest, throat, and sides, in one wild and ravaging sore.

"When I gave this advice, it was under the impression that the plan would be pursued under the immediate regulation of Mr. Green; and that I was only called upon to give advice. It was, however, soon decided, that the patient should go to Bedford, and be placed under my management. Previous to the undertaking, I thought it right distinctly to state my opinion of the case to a lady then present, and who had taken a very leading and anxious part in the business. In this representation it was frankly declared, that no promise more than of relief could be held out; but that, if the patient stil persisted in placing herself under my care, so far from shrinking from any effort, I would apply myself to the case with as much zeal and vigour as if an ultimate cure could be reasonably contemplated. The following is Mrs. Jennings own statement of the origin and progress of the disease.

"'In the year 1809, I first perceived a small lump in my breast, but thought little about it until the spring following, when it appeared somewhat enlarged, continued increasing in size, and became rather painful until June, when leeches were applied, which appeared to afford transient relief.

"'In 1811, the lump having become larger, though with little pain, I consulted Mr. Cooper, who ordered a frequent application of leeches, which appeared to occasion a contraction of the parts affected. After some months the drawn parts (under the nipple) began to ooze, and a few large red pimples appeared near the middle.

"'In 1812, I again shewed it to Mr. Cooper, when, upon saying I should not like to undergo an operation, as the arms appeared to be affected: he answered, it is very fortunate when the patient and surgeon agree; and added, he thought, from the breast being much lessened, it would probably quite disappear, when the complaint complaint might cease. He ordered leeches to be applied whenever the pain was troublesome, and gave me a prescription, which I followed until within the last half year. In the autumn of 1812, the red pimples broke, and have continued in a state of ulceration ever since.

" 'About two years and a half since, the other breast became affected, and has gone on in the same way as the first, except that it has been attended with more pain. In the summer of 1814, I shewed it to another surgeon in Town, who said *nothing* could be done; but added, when it was in great pain, I might put scraped carrot to the sores; and, upon my saying it never had been in much pain, he answered, perhaps not, but I must expect the pain to be extremely violent.

" 'It should be observed, that I had a very severe milk abscess of the left breast with my first child, now thirty-two years since. The whole breast was greatly diseased and swelled. The eschars, that were left, were deep and large. About nine years since, I had a violent blow on the same breast, from a butcher's boy in Leicester Fields. This, at the time, affected me so much, that I was obliged to go into a shop for relief. The pain, however, soon subsided, though at night it was again so painful as to induce me to rub it with brandy.

" 'In this state I have remained, with this only consolation in my mind, that some fever might release me, or that the bleeding from my breast might carry me off, before the threatened and multiplied horrors of the complaint overtook me.' "

Our limits only permit us to add the issue of this truly interesting case.

" June 12, 1815.—The bulk (says Mr. Young) of the minutes prevents their further publication at the present period; but it may be satisfactory to add, that this extraordinary and interesting case has now arrived so nearly to a completion of cure, that in the course of a week, in all human probability, there will not be a point of sore which is not covered by perfect and healthy cuticle.

" Throughout the progress, the astonishing provisions of nature have been established beyond all former record; and now, over the original sore of the chest, as well as that of the left breast, a large sheet of complete skin is spread, which has been formed and forming for the last three months. Mr. Short, one of the surgeons to the Bedford Infirmary, has had the satisfaction of witnessing the progress of this extraordinary cure, the details of which is circumstantially continued in the minutes of the case yet to be published."

" Being requested to state my own opinion of the improvement of my case since I have been under Mr. Young's care, I have only to say, such has been the wonderful change, and I am now so nearly well, that my return home is fixed for Monday in the next week. M. C. Jennings."

*Sunday, June* 11, 1815.

Such is the present state of this important change, we hope we may add, this important improvement, in the treatment of a disease the very mention of which has implied an incurable and painful local disease. We should add, that Mr. Whitbread's address has been properly attended to by the medical officers of the Middlesex Hospital. Indeed, from the invariable practice of that house, there can be no doubt that Mr. W.'s address, however well intended, was unnecessary; but it is with great satisfaction we can add, that the present trials encourage the further continuance of them.

---

*A Treatise on the best Management of Draught Horses in the Metropolis; especially those employed in the Coal Trade, in respect to their Purchase, Stabling, Dressing, Food, Labour, &c. And also on the Prevention and Treatment of their most fatal Diseases, contracted in the Stable, or in the Course of Labour.* By EDWARD HIGGS, Veterinary Surgeon for the Horse, and Member of the Royal Veterinary College, of Upper Thames-street. 18mo. pp. 129. London. Highley and Son, 1815.

THE title sufficiently explains the nature and subject of this little work. It only remains for us to observe, that the volume is likely to prove very beneficial to the public, and especially acceptable to all persons interested about horses. The author has had much experience in the veterinary art, but has chiefly devoted his attention to draught horses, an abused and suffering class of animals. Though his remarks are the result of extensive observation, which his large practice has enabled him to make, he has not omitted to consult other writers, and to select from them whatever is useful. His knowledge of anatomy, both human and comparative, and his acquaintance with the true principles of science, render his work more valuable than any that we have met with. "No man (he observes) can exercise this branch with credit to himself, or utility to the public, without a proper knowledge of anatomy. The veterinary surgeon labours under a disadvantage which does not apply to the human subject—his patient can give no information: he can only judge from appearances, and, unless he understand well the structure of the animal, the functions of every part, and the general harmony of the system in the sympathy of one part with another, he will often be at a loss to detect the presence and nature of disease; nay, not only must he trust to appearance, but he must often second this by examination, which can be of no avail without knowing minutely the structure structure and disposition of each part."—As the treatise is not strictly within our department, we shall not enter further into its consideration, but conclude with recommending it as a good practical performance.

---

*Edinburgh Medical and Surgical Journal*, No. XLIV, for October, 1815.

Art. I.—*Cases of Aneurism*. By John Mackesy, Surgeon 1st Batt. 62d Regt.

Case 1st was a Sicilian, aged 38, with a very large aneurism of the femoral artery.

"About a year from the period of my seeing him, while in the act of lifting a heavy forge-bellows, he suddenly felt a violent pain, and sense of tearing, on the inside of the right thigh, at the part where the artery perforates the tendon of the triceps: he immediately lost the power of supporting himself, and fell on the floor in great agony. On recovering from the first sensation, he examined the pained part, and discovered, that a pulsating tumour, about half the size of an egg, had already formed. On the tumour increasing in size, he was admitted into the principal hospital of Palermo, two months from the date of the accident; some months after he was removed from the chief hospital to that of Sant' Bartolemeo, an hospital appropriated to chronic and what are deemed incurable complaints."

Mr. Mackesy, under all the unfavourable circumstances of the limb being confined to a bent posture, and the low state of the patient's health, only deferred the operation to the day after he first saw him, partly encouraged by the importunity of the patient, and urged by the supplications of his partial friends and patrons.

"On the morning of the 8th of October, the operation was commenced by bringing the affected thigh into as direct a line with the pelvis as the situation of the limb would admit; a free incision, of about five inches in length, was then made through the integuments, commencing a little above Poupart's ligament, and continuing it down in the supposed track of the femoral artery, over the upper part of the base of the tumour.

"On dividing the integuments, a feeble pulsation of the artery could be felt at the upper part of the incision. On a few more strokes of the knife, a diseased mass of condensed cellular substance, of glands preternaturally enlarged, and of thickened fasciæ, were brought into view. The further steps of the operation were in some degree impeded by the blood thrown out by some twigs of the glandular arteries preternaturally enlarged. A cautious and tedious dissection at length laid bare the condensed cellular substance immediately including the artery, which being carefully opened, opened, and slit up for some space, the artery was without difficulty separated sufficiently from its attachments, with the handle of the scalpel; and, by means of the common eye-probe, a double ligature being introduced under it, one of them was tied two inches below Poupart's ligament, and the under one a little lower down, leaving a space of half an inch between them. The artery was now divided between the ligatures, and allowed to retreat within its surrounding cellular substance.

"The wound appeared now of a great depth, owing to the tumefaction of the part; and the course of the artery was somewhat removed from its natural direction, being forced more inward, by the vicinity of the tumour. In prosecuting the dissection, the utmost caution became necessary not to open the fascia at the lower point of the wound, as here it appeared to form the immediate covering of the superior part of the aneurismal tumour. The wound being cleared of blood, the integuments were accurately brought together, and secured by sutures and adhesive straps.

"The poor man bore the operation admirably, and his joy, on being informed that it was over, became so great, as to render it necessary to moderate it, by assuring him he was still in the utmost danger. The wound being dressed, the tumour was now examined; all pulsation had ceased; in other respects, the same as before. The patient, being laid in a bed made in the operating-room, was led to repose.

"9th October, morning visit.—Rested tolerably well during the night; pulse very little accelerated, and his skin of a natural temperature; the surface of the tumour was warmer than usual, and the foot and leg of the same temperature as the sound one. Low diet, and no medicines."

In the progress of the cure, the patient was seized with hectic symptoms, which gave an unfavourable appearance to the incised wound, and produced slough and suppuration in the aneurismal tumour. Notwithstanding all which, he recovered so far in the course of about ten weeks, as to be removed, in a state of convalescence, to the City Hospital.

The 2d patient was also a Sicilian, a native of Catania, subject to urinary calculi and other affections of the bladder and urethra.

"In the month of October, 1812, while endeavouring to raise a heavy copper boiler, he felt something tear, or give way, in the right groin, attended with extreme pain. On examining the pained part, he perceived a small pulsating tumour had formed, about the size of an almond, which gradually increased in size; its advance, he conceives, has been considerably forwarded by his straining to make water.

"I saw him, on the 15th of October, 1813, in a ward of the chief hospital of Palermo, and the following appearances were observable:—A large oblong pulsating tumour occupied the right groin groin and inside of the thigh; its base extended from the spinous process of the ilium to within six inches of the knee; its apex rather on the front of the thigh; and, about four inches below Poupart's ligament, a turgid branch of a vein run along its surface, which was painful, and covered with an erysipelatous blush of inflammation. The pulsation of the tumour was so strong, as to render the raising and falling of the bed-clothes perceptible at each stroke of the artery; the foot and leg of the affected limb were œdematous, but of a natural temperature; the pulsation was most distinctly felt immediately under the femoral ligament, and at this point each pulsation gave to the finger a sort of jarring sensation; a low irritable fever was present, with quick wire-like pulse, 126 in the minute; great emaciation had evidently occurred, which, with a sunk eye, and general cadaverous aspect, forcibly declared his miserable situation.

"His medical attendants, viewing him, as it were, already in the grave, ceased their unavailing attendance. Extreme unction has been four times administered to him; and he has daily at his bedside a priest, preparing him for another world.

"The case was clearly an aneurism of the femoral artery, the vessel being ruptured where it passes over the brim of the pelvis, and under the ligament of Poupart. The complaint was of twelve months' duration, and was deemed by the attending medical officers of the establishment as incurable; the patient was, of course, left to a certain fate.

"I proposed to one of the principal physicians of the hospital (Dr. Calcagni), that there still existed a possibility of saving the patient by an operation, although, from his advanced age and unfavourable situations, the result would be very doubtful. This gentleman, in concurrence with the sub-director of the hospital, most readily agreed to an expedient which even held out remote hopes of success. The patient himself most anxiously begged that the operation might be performed immediately.

"Permission being obtained for his removal into the British detachment hospital, he was removed there on the evening of the 16th of October, 1813. On the morning of the 17th the operation was to be performed, but we were obliged to defer it till next day, in consequence of the height of the fever and extreme agitation of the patient.

"From the ease derived from a relaxed state of the muscles, the patient, for months before, kept the thigh bent towards the abdomen; the increasing size of the tumour fixed it in this position, so that at this period the thigh could not be stretched out on a line with the pelvis. The superior spine of the ilium could not be distinctly felt, in consequence of the base of the tumour extending over it. The ligament of Poupart was pressed upwards, and considerably elevated, crossing like a band the upper part of the tumour.

"18th of October.—The patient being now more tranquil, although though the fever continued, with a pulse of 120 in the minute, further delay was deemed hazardous. Being favoured with the very obliging assistance of Surgeons Shortt, Prichard, and Boltan, the operation was commenced, in presence of a numerous body of the city surgeons and physicians.

"An incision was made through the integuments, commencing two inches above the superior spinous process of the ilium, and about one and a half inch from it, and terminating at that point of Poupart's ligament, where the pulsation appeared most distinct; its direction downwards was sufficiently removed from the abdominal ring, as to pass on the outside of its superior internal opening, thus avoiding the hazard of wounding the spermatic chord, or epigastric artery. The first incision brought into view the cellular substance covering the tendon of the obliquus externus; this substance was much firmer than natural, assuming the appearance of a yellow tendinous expansion. This, with the tendon of the oblique muscle, being dissected through, and slit down to the ligament, the internal oblique was laid bare.

"From the morbid thickness which the parts had acquired, it became necessary to divide some fibres, before the finger could be admitted between Poupart's ligament and the edges of the obliquus internus and transversalis. This being carefully done, I was now enabled to introduce the fore-finger of the left hand between these muscles and the peritonæum, which formed a director to the bistoury, with which I divided them in the direction, and nearly to the extent, of the external wound.

"From the flaccid state of the abdomen, owing to the emaciation of the patient, it only became necessary to disturb the peritonæum in a slight degree; but the access to the external iliac artery was impeded by a confused mass of glands and cellular substance. These being separated, and held aside, the artery was discovered free of the tumour, and pressed inwards on the psoas parvus. Some fibres of the fascia iliaca, where it formed a sheath to the crural vessels, were divided between the nerve and artery. With the handle of the scalpel, the artery was now sufficiently detached. A blunt needle, with a double ligature, was, without difficulty, passed beneath it. Each ligature being tied, the artery was divided between them, about two and a half inches above Poupart's ligament. On the artery being tied, all pulsation ceased in the tumour; its surface, a moment before red and inflamed, became immediately pale, with diminution of temperature; the entire limb likewise became colder than usual a few moments after. The integuments were now brought together, and retained by sutures and adhesive plaster. A bed being made in the room where the operation was performed, he was placed in it, and left in charge of a careful attendant."

These cases do great honour to British surgery and surgeons, and shew, that notwithstanding the just celebrity of Scarpa, whom we may partly claim as an *alumnus* of London, don, the progress of the art is very slow in Italy, either from the little encouragement it receives, or from the paucity of establishments, and the expence attending a migration to, and a short residence at, Pavia. On the operations, we shall only remark, that, as nothing could be more successful, so we conceive nothing could be more judicious. Respecting the cases, we feel obliged to the author for all the previous history he could collect, but hope, after the endeavours of Scarpa, and the addition which the industry and genius of Mr. Hodgson have made to this part of surgery, that the origin of such complaints will be by degrees better understood. Though the patients were first sensible of the pain, tumour, and pulsation, after violent muscular exertion, yet we are far from imputing the origin of the disease to that cause. Probably the artery had been diseased long before, and only then gave way in its internal coat. These subjects must, we conceive, be soon better understood.

Art. II.—*Case of Amaurosis cured by Active Treatment.* By John Bishop Estlin, Member of the Royal Medical Society, Edinburgh, and of the Royal College of Surgeons, London.

Our readers will recollect the success ascribed by Mr. Stevenson to depletion in some cases of morbid sensibility of the eye, which have too often been considered as nervous, and treated in a very different manner. Mr. Estlin's case is very pointed and very interesting, and might, without any disadvantage, have been given more at length. The patient, a female, only eight years of age, had previous symptoms of plethora or congestion about the head. On rising in the morning, her eyes had been so much affected during the night, that she was insensible to the light of the day. The mode of treatment was judicious as to the evacuations, but we suspect the author would have found more advantage from topical bleeding than the application of a blister to the head. This can only be meant as a hint, which we are not afraid will be well received by one who writes with such candour.

"On inquiring (says Mr. Estlin) of the parents of the child respecting the prevailing disorders in their family, I found that they had lost *five* children by some disease of the head, of which the most striking symptom to their observation was convulsions. The mother recollected, when I particularly asked her, that the subject of these remarks had for some time complained of head-ach, though it was not sufficient to prevent her from attending regularly the Lancastrian girls' school in this city, of which she was a pupil. Her bowels were, in general, rather constipated. These circumstances, together with the severity of the attack, made me apprehensive hensive that the optic nerves were affected with a greater degree of disease or pressure than it was in the power of medical treatment to relieve."

It would have added to the interest of the case, if Mr. Estlin had informed us at what age the other children were attacked, and whether such as survived that period continued to be healthy, or free from those particular complaints. These inquiries may be highly important to any future offspring of the same parents.

Art. III.—*Case of Hydrophobia, unsuccessfully treated by copious Bleeding.* By Dr. Albers, of Bremen.

The case is very candidly related. The quantity of blood taken was considerable, and the first venesection only about six hours after the symptoms commenced. There was, however, a strong previous conviction in the mind of the patient that she must die, and, contrary to her own inclination, she was forced into the Infirmary. In the course of the day she had lost 80 oz. of blood. We shall transcribe the concluding scene with only a single remark.

"In spite of her excruciating thirst, she could not swallow a single drop; and the word *drink* alone was now sufficient to produce much more violent convulsive tremors in all her frame. The pills and some bread she swallowed without any difficulty. The pulse was quick, yet not so low as to make me think I durst not venture on a repeated bleeding. When twenty ounces of blood had been drawn from her right arm, she nearly fainted, but soon recovered, and was put to bed. Soon after I had withdrawn, at eleven o'clock, she experienced a still greater oppression and anxiety, started from the bed, ran to and fro in the room, and from time to time cried out so, that her cries were heard at the distance of several houses from her habitation. She repeatedly warned her attendant to keep at a distance, that she might not bite him. She said that she was an unfortunate creature, and did not wish to bring her misfortune upon others. I am still uncertain whether she actually felt this propensity to bite or not, (though I have witnessed the same symptom with another patient at the great hospital at Vienna,) and rather suspect that she had formerly been told, that people bit by mad dogs had an inclination also to bite. The terrible thirst she suffered forced her, from time to time, to dip her finger in the water-gruel, and moisten her tongue, which, as the nurse asserted, did, however, not increase the paroxysms of her anxieties. She now, likewise, felt such an aversion from the swallowing of the pills, that they must be put in her hand, while she laid on her back, and then, with her eyes shut, conveyed them to her mouth. Till two o'clock in the morning, the paroxysms of anxiety, from time to time, subsided. After that time, however, they continued, without intermission, till four o'clock, when the patient patient of a sudden became calm, dropped down, and expired. At three o'clock she had sent for me, with the request, as she was no more able to bear her oppression and anxiety, to bleed her to death; which, as well may be thought, was not complied with. Instead of this, I prescribed pills, each of which contained one grain of ipecacuanha, and one grain of opium. On their arrival, the patient was no more."

As, during the latter period, there were still symptoms of great anxiety, considerable action, and, probably, strength, would it not have been admissable to have repeated the bleeding? The proposal of bleeding her to death, we do not consider as any apology for so doing; but the relief she had experienced at each venæsection, and the certainty of death if the then symptoms continued, might, we conceive, have been a sufficient indication of the *remedium ancepts* of Celsus.*

Art. IV.—*Brief Hints, relative to the Improvement of the Pathology and Treatment of those Chronic Diseases usually termed Nervous.* By JOHN ARMSTRONG, M.D. Sunderland.

Lord Chesterfield cautions his son against *local* wit, which, he observes, may be vapid in any other spot. We would caution certain writers against local " hints." " Every practitioner," says Dr. Armstrong, " must be aware how vaguely we use medical language with regard to the term *Neuroses*." In confirmation of this, we have only to urge, as we have often done, the injury which medicine has sustained in many other instances, by the too early attempts of nosologists. But this injury has been greatly increased by the just celebrity, in other respects, of the northern school; and by the nosological table becoming the text book of academic lectures. The injury to practice was, however, short-lived in the south, and has long since ceased to exist, we believe, every where; so that Dr. Armstrong's hints need not, as he addresses them, be directed to *every* practitioner.

Art. V.—*Letter on the Treatment of Tetanus, to Dr. Dickson, Physician to the Fleet, from* Mr. E. GRIMSTONE, Surgeon to his Majesty's Ship the Tonnant.

This is a very judicious paper. The author divides tetanus into three varieties—the acute; the chronic from local injury; and the chronic idiopathic, or arising without any local injury. All these, he remarks, are most common in warm climates.

---

* For a more successful issue from blood-letting in hydrophobia, see our Intelligence.

We conceive our author might have ventured on making two *species*, and dividing the last into two *varieties*. Our reason for mentioning this arises from his own well-founded observation, that the acute is, for the most part, the only dangerous form. The difference cannot be too strongly insisted on, because, for want of it, so much confusion has arisen in the application of remedies. In our late remarks on Dr. Perry's valuable treatise on this subject, we gave that gentleman the credit of being the first among the moderns to repeat an observation overlooked since the time of Celsus, viz. that the acute form of the disease usually proves fatal on the third day.

"Of the last (the chronic) state of the disease," says Mr. G. "the greater number of cases, I think, do well; of the acute branch, depending on local injury, which mostly takes place where the wound (let it be great or small) is in a highly painful and irritable state, I am led to believe, there is scarcely an instance of recovery, it generally proving fatal within the third day, in spite of all the efforts of medical aid. But it is the chronic state of the symptomatic disease in which I have been successful, and which, I feel satisfied, is what we so often hear of yielding. Here it may be remarked, that the two first species of the disease are in a great measure distinct as to the period of their attack, the acute making its appearance in a short time after the infliction of the wound, and, as I have observed above, when it is in a highly painful and irritable state; and the chronic seldom or ever taking place, until the inflammatory symptoms have subsided, or the wound is far advanced in the cure, and often many days, after it has entirely healed. See Miller's case. Stimulants have done the most service in these cases; of this we have the most unequivocal evidence."

"A volume of evidence," we are afterwards told, "might be produced, to prove the pre-eminence of stimulants in the cure of the milder tetanic affections, in proof of which I refer you to the latest publications of the day on that disease.

"Although it will be differing from so respectable an authority as M. Larrey, yet I must say, I have never seen any permanent relief, either from local applications, or removal of parts. Amputation was performed in several cases which came under my knowledge during my service in India, and in two cases of my own, while surgeon of the hospital-ship at Prince of Wales' Island, both symptomatic, and of the acute kind. The operation was performed on the first appearance of the disease, the difficulty of swallowing increased hourly, and they both died within the third day. Opium was administered in various shapes, and in large quantities, without the smallest effect.

"Your valuable hints I shall not refer to at present; farther, than not being able altogether to coincide with you, that bleeding occasionally, and purgatives, might be a means of preventing the disease, disease. However salutary bleeding might be in the early symptoms of one species, I feel confident it would prove injurious in that of which I mean more particularly to speak. Dr. Rush observes, 'If I may be allowed to judge of the probable effects of blood-letting, from what I have seen to follow its use in tetanus, it will be better omitted.'

"But, without touching upon the importance you justly ascribe to a full and natural performance of the intestinal functions, in diseases in general, and to the free and healthy condition of the alvine discharges, as tending to obviate that in question, I sincerely hope your expectations of the utility of purgatives, if not for the cure, at least for the prevention of tetanus, 'unless where the patient is exposed to the strongest exciting causes,' may be realised; for I have always found the torpid state of the bowels, when once the disease has taken place, whether arising from the disease itself, or from the remedies employed, forms one of the most troublesome and difficult branches of the cure.

"It appears to me, that the most successful mode of treating this disease hitherto has been by creating and keeping up, for a certain period, a strong action in the system, by some powerful stimulus, greater than the morbid one already existing; but whatever is likely to promise success in this dreadful malady, should be encouraged and tried in every shape, and in every part of the globe. To look with confidence even on one of its branches, would be gaining a great point, and be relieving the mind of the patient, as well as the surgeon. I have known many refuse to allow amputation where it was likely to have saved life, (in an hospital,) from no other reason than the fear of 'locked-jaw.'

"I am disposed to think, that, if the fifth day could be got over, we might promise ourselves success. As a general circumstance, I may state, that, in three cases of amputation of the lower extremities, among the wounded of the Dutch frigate Pallas, two, where the needle was used, had the disease, and died within the third day; and the other, where the arteries were taken up, principally with the tarnaculum, did well, without the slightest symptom of it."

Two cases are very minutely related. We transcribe the last, with the author's concluding remarks.

"Mr. Charles Wattell, clerk of His Majesty's brig Diana, aged about 30 years, of a spare habit, was received into H. M. hospital-ship, Wilhelmina, on the 20th May 1809, for the cure of a wound caused by a pistol ball passing through the palm of the right hand. The integuments were much lacerated, and the middle metacarpal bone considerably shattered.

"Upon first viewing the case, I advised amputation above the wrist, giving him my reasons for so doing, founded on the great probability of a locked-jaw taking place, particularly in this climate, but he would not consent. I then dissected out both parts of the metacarpal bone with the finger, and brought the integuments ments together, on both sides of the hand, by stitches, applied soft dressings, and gave extract. opii gr. ij.

"22d.—Considerable heat; costive; hand and arm very painful.

℞. Mist. nitros. ℥vj.
Tinct. opii, gtt. xxx. ft. M. capiat. cochlear. duo tert qq. hor.
*Vesp.* Extract. opii gr. ii. et cras mane sol. Sal. Glaub. ℥iij. which had the desired effect.

"24th.—Much less pain; pulse moderate; skin cool; and the bowels open. Dressed the wound, which looked and discharged well.

℞. Pulv. cinchonæ ℥ſs.

Spt. vini Gall. ℥iſs.

Aquæ puræ ℥viſs. ft. mist.

Sumat ℥i. tert. qq. hor. *Vesp.* Ext. opii gr. ii.

"He continued to do well until the 8th of June, when the wound was nearly healed, and I observed signs of tetanus, such as difficulty of swallowing, turning the head, alteration of speech, and the jaw slightly contracted. On inquiry, I found he had general spasms for the two last days past, particularly violent about the neck and back, which he had neglected to tell me of before. I gave him tinct. opii gtt. xx. statim, and repeated ten more every hour. Again pressed him to submit to amputation, but he still refused; consequently I applied a pledget, dipped in equal parts of tinct. opii et spts. terebinth. to the wound.

"June 9th.—The jaw completely set, so as not to admit of fluids but with difficulty; spasms general and violent; no stool since the 7th instant.

℞. Tinct. opii ʒi.

Aquæ puræ ℥viij. Capiat ℥i, omni hora. Put him into the cold bath.

"10th.—No alteration. Continued the mixture as before, with tinct. opii ʒiſs. et tinct. digitalis ʒſs.

℞. Calomel. ppt. gr. viii. statim sumend. *Vesp.* Solut. sal Glaub. ℥iiſs.

"11th, 12th, and 13th.—Spasms extremely distressing; costive. Ol. ricini ʒx. statim.

℞. Tinct. opii ʒij.

Aquæ puræ ℥viij. M. Capt. ut antea.

*Vesp.* Bowels open. Cont. mist.

"14th.—I put ʒiiſs. tinct. opii to the mixture, and on the 17th ʒiii. during which time his sufferings were very great. I had kept the bowels open. Wound healed. This morning the spasms were frequent, but certainly not so violent. I now, from the length of time, and the diminished state of the symptoms, began to entertain some hope. Continued the medicine, as above, till the 24th, without any material alteration. Pulse generally quick, and inclined to fulness. Previous to my visiting him this evening, my assistant told me that the spasms had entirely gone off. On my going to him, I found the jaw much relaxed, and he said he was free from

4 pain, pain, but to all appearance much intoxicated; and, on further inquiry, I found he had been very much so in the early part of the day. I directed the mixture to be continued as usual.

"25th.—The spasms were general, violent, and the jaw contracted more than ever; had one stool; pulse not so full; profuse perspiration; and complained of languor. I continued the mixture, as above, every hour, and gave Extract opii gr. i. every two hours, and rather better than an ounce of good spirits *every hour*. I continued this until the 28th, when he was evidently better. Cont. med.

"July 1st.—Still better; could take slightly of solids, and use the jaw without bringing on the spasm.

℞. Ol. ricini ℥x. statim. Cont. mist.; and gave gr. ii. of the extract of opium every second hour.

"From this day he gradually recovered. I made no alteration in his medicines, excepting omitting a part of the spirits, till the 10th, when he could open the mouth, and make use of the jaw as well as ever. The debility was great, and now and then a slight spasm takes place, from the right shoulder to the temple of the same side; but it diminishes daily.

"During the progress of the disease, I observed the quantity of opium given neither affected the head nor procured sleep. He generally complained of want of rest. The tincture was made by myself, and very good. I took the idea of preferring it to the extract from the writings of Professor Mursinna, of Berlin. I also observed, in the use of the cold-bath, that, although the interval was somewhat lengthened, the spasm followed with greater force; and I am much in doubt whether the digitalis had not a similar effect.

"Whether may we attribute the success of the case to the stimulus kept up by the spirits, or to the antispasmodic effects of the opium, I am at a loss to know. I should rather conclude it was the combined action of both; and although deserving of further trial, it would be dangerous to depend on the spirits alone.

"I must further observe, that my patient was one who indulged freely in the use of spirits when in health; consequently, we might expect its stimulating qualities would have less effect on him than on one who had not done so."

Art. VI.—*Observations on the Treatment of Gun-shot Wounds*. By George Power, Surgeon to the Forces.

(To be continued.)

Medical

